# Circulating Biomarkers and Cardiac Structure and Function in Rheumatoid Arthritis

**DOI:** 10.3389/fcvm.2021.754784

**Published:** 2021-11-18

**Authors:** Masatake Kobayashi, Maria Betânia Ferreira, Rita Quelhas Costa, Tomás Fonseca, José Carlos Oliveira, António Marinho, Henrique Cyrne Carvalho, Nicolas Girerd, Patrick Rossignol, Faiez Zannad, Patrícia Rodrigues, João Pedro Ferreira

**Affiliations:** ^1^Université de Lorraine, INSERM, Centre d'Investigations Cliniques Plurithématique 1433, INSERM U1116, CHRU de Nancy and F-CRIN INI-CRCT, Nancy, France; ^2^Unit of Multidisciplinary Research in Biomedicine, Porto, Portugal; ^3^Instituto de Ciências Biomédicas Abel Salazar, School of Medicine and Biomedical Sciences, University of Porto, Porto, Portugal; ^4^Hospital da Luz Arrábida, Porto, Portugal; ^5^Internal Medicine Department, Centro Hospitalar de Entre o Douro e Vouga, Aveiro, Portugal; ^6^Internal Medicine Department, Centro Hospitalar Universitário Do Porto, Porto, Portugal; ^7^Clinical Chemistry Service, Centro Hospitalar Universitário Do Porto, Porto, Portugal; ^8^Cardiology Department, Centro Hospitalar Universitário Do Porto, Porto, Portugal; ^9^Department of Surgery and Physiology, Cardiovascular Research and Development Center, Faculty of Medicine of the University of Porto, Porto, Portugal

**Keywords:** rheumatoid arthritis, heart failure with preserved ejection fraction, echocardiogram, circulating biomarkers, prognosis

## Abstract

**Background:** Rheumatoid arthritis (RA) increases the risk for abnormalities of the cardiac structure and function, which may lead to heart failure (HF). Studying the association between circulating biomarkers and echocardiographic parameters is important to screen patients with RA with a higher risk of cardiac dysfunction.

**Aim:** To study the association between circulating biomarkers and echocardiographic parameters in patients with RA.

**Methods:** Echocardiography was performed in 355 patients with RA from RA Porto cohort and the associations between echocardiographic characteristics and 94 circulating biomarkers were assessed. These associations were also assessed in the Metabolic Road to Diastolic Heart Failure (MEDIA-DHF) [392 patients with HF with preserved ejection fraction (HFpEF)] and the Suivi Temporaire Annuel Non-Invasif de la Santé des Lorrains Assurés Sociaux (STANISLAS) (1,672 healthy population) cohorts.

**Results:** In the RA Porto cohort, mean age was 58 ± 13 years, 23% were males and mean RA duration was 12 ± 10 years. After adjustment and multiple testing correction, left ventricular mass index (LVMi), left atrial volume index (LAVi), and E/e′ were independently associated with biomarkers reflecting inflammation [i.e., bone morphogenetic protein 9 (BMP9), pentraxin-related protein 3 (PTX3), tumor necrosis factor receptor superfamily member 11a (TNFRSF11A)], extracellular matrix remodeling [i.e., placental growth factor (PGF)], congestion [i.e., N-terminal pro-brain natriuretic peptide (NT-proBNP), adrenomedullin (ADM)], and myocardial injury (e.g., troponin). Greater LVMi [hazard ratio (HR) (95% CI) per 1 g/m^2^ = 1.03 (1.02–1.04), *p* < 0.001], LAVi [HR (95% CI) per 1 ml/m^2^ = 1.03 (1.01–1.06), *p* < 0.001], and E/e′ [HR (95% CI) per 1 = 1.08 (1.04–1.13), *p* < 0.001] were associated with higher rates of cardiovascular events. These associations were externally replicated in patients with HFpEF and asymptomatic individuals.

**Conclusion:** Circulating biomarkers reflecting inflammation, extracellular matrix remodeling, congestion, and myocardial injury were associated with underlying alterations of cardiac structure and function. Biomarkers might be used for the screening of cardiac alterations in patients with RA.

## Introduction

Rheumatoid arthritis (RA) is a chronic condition characterized by systemic inflammation affecting nearly 1% of the population (1). As a consequence of the underlying proinflammatory and profibrotic state, patients with RA have a high risk of progressing toward cardiac structural and functional abnormalities [e.g., left ventricular hypertrophy (LVH), left atrial enlargement, and diastolic dysfunction] (2, 3). These cardiac alterations may increase the risk of developing heart failure (HF) (4).

Circulating biomarkers may provide relevant information about the underlying pathophysiological processes associated with both the RA and cardiac alterations, even when asymptomatic (5, 6). Determining the circulating biomarkers that correlate with the cardiac structure and function of patients with RA may help to better identify individuals at a risk of developing symptomatic HF and implement biomarker-based screening strategies. In this regard, consistent associations between inflammatory biomarkers and abnormal diastolic function were reported in HF with preserved ejection fraction (HFpEF) likely because HFpEF is a condition characterized by high expression of inflammatory markers (4, 7).

The RA Porto cohort (ClinicalTrials.gov Identifier: NCT03960515) provides a good opportunity to explore the circulating biomarkers associated with echocardiographic parameters in RA and the prognostic value of echocardiographic parameters (8). Additionally, the associations between biomarkers and echocardiographic parameters were also assessed in a cohort of patients with HFpEF of the Metabolic Road to Diastolic Heart Failure (MEDIA-DHF) cohort study (ClinicalTrials.gov identifier: NCT02446327) (9) and a cohort of an initially healthy population in the *Suivi Temporaire Annuel Non-Invasif de la Santé des Lorrains Assurés Sociaux* (STANISLAS) (ClinicalTrials.gov identifier: NCTO1391442) (10).

## Methods

### Study Population

This study is reported as per the Strengthening the Reporting of Observational Studies in Epidemiology (STROBE) guideline (Supplementary Table 1). The RA Porto cohort included 408 patients aged ≥ 18 years with RA diagnosis according to the 2010 American College of Rheumatology/European League Against Rheumatism (ACR/EULAR) criteria (11) followed in the Autoimmune Disease Unit of *Centro Hospitalar Universitário do Porto*, Portugal from June 2016 to June 2018 as previously published (8). Demographic parameters, medical history, physical examination, laboratory findings and treatments, and RA-related information (i.e., RA duration period) were collected. Patient median follow-up time was 1,459 days (4.0 years). Cause of death and hospitalization were independently adjudicated. For the current analysis, we included patients with available data on echocardiogram and biomarkers. This study was conducted in accordance with the Declaration of Helsinki and approved by the Institutional Ethics Committees and all the participants signed a written informed consent prior to entry into this study.

The MEDIA-DHF cohort study included 626 patients with HFpEF diagnosis according to the 2007 European Society of Cardiology (ESC) consensus recommendations between 2012 and 2014 as previously published (9). A total of 392 patients with HFpEF with available data on echocardiographic parameters and biomarkers were included.

The STANISLAS cohort study is a single-center familial longitudinal population-based cohort from the Nancy region in France as previously reported (10). Among 1,705 participants undergoing the 4th cohort visit, 1,679 participants had available both the echocardiographic and biomarker data.

The data used for this report can be assessed by other authors upon reasonable request to the corresponding author.

### Echocardiography

Echocardiographic parameters were acquired by one cardiologist that was blinded to clinical data and circulating biomarker values of patient by using the Philips® iE33 Ultrasound Machine, Philips, Bothell, WA. Cardiac chamber and systolic function [i.e., left ventricular ejection fraction (LVEF), left ventricular mass index (LVMi), left atrial volume index (LAVi), and inferior vena cava (IVC) diameter] were assessed according to the international recommendations (12, 13). Pulmonary arterial systolic pressure (PASP) was estimated by using peak tricuspid regurgitation (TR) velocity and IVC compliance and diameter (13). Diastolic function was assessed from the mitral inflow pattern by pulsed-wave Doppler. Mitral annular early diastolic velocity (e′) was assessed at the septal and lateral sites of the mitral annulus by using tissue Doppler imaging. E/A ratio, e′ mean, and E/e′ mean ratio were calculated (14). LVH was defined by a LVMi > 115 g/m^2^ in males or > 95 g/m^2^ in females (12). The measurement reproducibility is shown in Supplementary Table 2.

### Circulating Proteomic Biomarkers

A large biomarker panel with 92 biomarkers from a wide range of pathophysiological domains was measured (Olink® CVDII panel). An overview of biomarkers, their full names, UniProt ID, and roles are presented in Supplementary Table 3. These biomarkers were measured by using a high-throughput technique by using the Olink Proseek® Multiplex CVDII 96 ×96 kit, which measures 92 biomarkers simultaneously in 1-μl plasma samples. The kit uses a proximity extension assay (PEA) technology where 92 oligonucleotide-labeled antibody probe pairs allowed to bind to their respective target present in the sample. Biomarker expression is provided in a Log2-normalized scale, Normalized Protein eXpression (NPX). For more details, please go to http://www.olink.com/ (15). In addition to the Olink® CVDII panel, in the RA Porto cohort, N-terminal pro-brain natriuretic peptide (NT-proBNP) and high-sensitivity troponin T (hsTnT) were measured by the Elecsys (Roche Diagnostics®, GmbH, Penzberg, Germany) NT-proBNP and troponin T (Gen 5 STAT test). In the MEDIA-DHF and the STANISLAS cohorts, NT-proBNP and high-sensitivity troponin I (hsTnI) were, respectively, measured by the Olink® CVDIII and organ damage panel.

### Statistical Analysis

Categorical variables are described as frequencies (percentages), while continuous variables are described as mean ± SD or median (25th and 75th percentiles) depending on the variable distributions. The multivariable linear regression analysis was performed to test the associations between the circulating biomarkers and echocardiographic parameters. Models included relevant confounders as previously shown (3, 16): age, sex, body mass index (BMI), diabetes, systolic blood pressure, estimated glomerular filtration rate (eGFR) [the Chronic Kidney Disease Epidemiology Collaboration formula (17)], and RA duration. In the MEDIA-DHF and the STANISLAS cohorts, the above confounders excluding RA duration were adjusted to assess the associations between biomarkers and echocardiographic parameters. Multiple testing correction for false discoveries was set at 5% (false discover rate (FDR) <0.05), as described by Benjamini and Hochberg (18).

In the RA Porto cohort, the primary outcome was the composite of cardiovascular mortality or hospitalization for cardiovascular reasons. Hospitalization for cardiovascular events included HF hospitalization, myocardial infarction, acute coronary syndromes, angina pectoris, stroke, transient ischemic attack, and peripheral artery diseases. Survival probabilities were estimated by using the Kaplan–Meier method. The Cox proportional hazards model for echocardiographic structure/function abnormalities was used to obtain the unadjusted and covariate adjusted hazard ratios. In addition, we explored the associations of biomarkers with the outcome after adjustment for echocardiographic parameters plus clinical covariates. No data imputation was performed.

All the statistical analyses were performed by using the R software version 4.0.1 (R Development Core Team, Vienna, Austria, UK).

## Results

### Baseline Characteristics

The mean age of the 355 patients with RA included in the RA Porto cohort was 58 ± 13 years, 23% were males, 20% were obese (BMI ≥ 30 kg/m^2^), and 47% had hypertension. The mean RA duration of these patients was 12 ± 10 years, 34.6% had an erosive disease, and 45.6% took corticosteroids. The mean LVEF was 61 ± 7%, 9.0% had LVH based on LVMi, 36% had left atrial enlargement (LAVi ≥ 34 ml/m^2^), and 8.4% had elevated E/e′ ratio (E/e′ > 14) (Table 1).

**Table 1:** Baseline characteristics in the RA Porto cohort (*N* = 355).

	**Mean ± SD or *N* (%)**
Age, yrs	58.4 ± 13.1
Male, *N* (%)	84 (23.1%)
Body mass index, kg/m^2^	26.6 ± 4.5
**Medical history**, ***N*** **(%)**	
Hypertension	170 (46.8%)
Diabetes	51 (14.0%)
Dyslipidemia	174 (47.9%)
Coronary artery disease	8 (2.2%)
Heart failure	115 (32.4%)
Atrial fibrillation	13 (3.6%)
Chronic obstructive pulmonary disease	18 (5.0%)
Smoking	51 (14.0%)
NYHA > III	28 (7.7%)
Systolic blood pressure, bpm	132.9 ± 19.0
Heart rate, bpm	80.6 ± 14.6
**RA history**	
RA diagnostic (years)	11.5 ± 10.1
RF or anti CCP positive, *N* (%)	277 (78.0%)
Articular erosions, *N* (%)	123 (34.6%)
DAS28 VS (ESR)	2.8 ± 1.2
DAS28 VS (CRP)	2.4 ± 1.1
**Medication**	
ACE inhibitor or ARB, *N* (%)	136 (38.3%)
Beta-blocker, *N* (%)	40 (11.3%)
Calcium channel blocker, *N* (%)	40 (11.3%)
Aldosterone antagonist, *N* (%)	2 (0.6%)
Loop diuretics, *N* (%)	10 (2.8%)
Statin, *N* (%)	134 (37.7%)
Corticosteroids, *N* (%)	162 (45.6%)
Methotrexate, *N* (%)	215 (60.7%)
NSAIDs, *N* (%)	85 (23.9%)
Biological DMARDs, *N* (%)	65 (18.3%)
Individual DMNARDs	
Anti-TNFα	38 (60.3%)
Rituximab	11 (16.2%)
Tocilizumab	16 (23.5%)
**Biochemistry**	
HbA1c, %	5.6 ± 0.8
LDL cholesterol, mg/dl	101.0 ± 31.0
Hemoglobin, g/dl	13.1 ± 1.4
eGFR, ml/min/1.73 m^2^	88.3 ± 20.3
**Echocardiogram**	
LVEF, %	61.0 ± 7.1
LVEF <50%, *N* (%)	17 (5.0%)
LVMi, g/m^2^	69.6 ± 21.4
LV hypertrophy, *N* (%)	32 (9.0%)
LAVi, ml/m^2^	32.4 ± 10.9
LAVi >34 ml/m^2^, *N* (%)	129 (35.7%)
E/A ratio	1.0 ± 0.7
e′ mean, cm/s	8.9 ± 2.7
Septal e′ <8 cm/s or l ateral e′ <10 cm/s, *N* (%)	205 (57.7%)
E/e' mean	9.3 ± 3.9
E/e′ mean >14, *N* (%)	30 (8.4%)
Peak TR velocity, cm/s (*N* = 178)	2.3 ± 0.4
Peak TR velocity > 2.8 cm/s, *N* (%) (*N* = 178)	12 (6.7%)
Pulmonary artery systolic pressure, mmHg	23.7 ± 7.6

*Values are mean ± SD, n (%) or median (25th−75th percentile).*

*NYHA, New York Heart Association; RA, rheumatoid arthritis; anti-CCP, anti-citrulline antibody; DAS28 VS (ESR), Disease Activity Score for Rheumatoid Arthritis with Erythrocyte Sedimentation Rate; ACE inhibitor, angiotensin converting enzyme inhibitor; ARB, angiotensin receptor blocker; NSAIDs, nonsteroidal anti-inflammatory drugs; DMARDs, disease-modifying anti-rheumatic drugs; HbA1c, glycosylated hemoglobin; LDL, low-density lipoprotein; eGFR, estimated glomerular filtration rate; LVEF, left ventricular ejection fraction; LVMi, left ventricular mass index; LAVi, left atrial volume index; TR, tricuspid regurgitation*.

### Association of Circulating Biomarkers With Echocardiographic Parameters

The circulating biomarkers associated with echocardiographic parameters are shown in the Supplementary Tables 4–6.

In patients with RA, LVMi was positively associated with higher levels of hsTnT and BNP/NT-proBNP and negatively associated with bone morphogenetic protein 9 (BMP9); LAVi was positively associated with BNP/NT-proBNP, interleukin-4 receptor subunit alpha 4 (IL4RA4), T-cell surface glycoprotein cluster of differentiation 4 (CD4), and thrombospondin 2 (THBS2); E/e′ was positively associated with hsTnT, BNP/NT-proBNP, adrenomedullin (ADM), placental growth factor (PGF), spondin-2 (SPON-2), THBS2, tumor necrosis factor receptor superfamily member 11a (TNFRSF11A), pentraxin-related protein 3 (PTX3), cathepsin L1 (CTSL1), and CD4 (FDR, *q* < 0.001) (Table 2).

**Table 2:** Multivariable selected biomarkers for each echocardiographic parameter in the RA porto cohort.

**LVMi (per 1 g/m** ^**2**^ **)**				**LAVi (per 1 ml/m** ^**2**^ **)**				**E/e**^**′**^ **mean (per 1)**			
**Biomarkers**	**Beta (95%CI)**	***p*-value**	**FDR**	**Biomarkers**	**Beta (95%CI)**	***p*-value**	**FDR**	**Biomarkers**	**Beta (95%CI)**	***p*-value**	**FDR**
KIM1	4.41 (1.74 to 7.08)	<0.001	0.02	SLAMF7	2.59 (1.03 to 4.16)	0.001	0.02	ADM	1.64 (0.77 to 2.51)	<0.001	0.01
BMP9	−6.30 (−9.88 to −2.72)	<0.001	0.01	IL4RA	5.71 (2.51 to 8.91)	<0.001	0.01	PGF	2.63 (1.56 to 3.7)	<0.001	<0.001
Troponin	7.25 (3.87 to 10.62)	<0.001	<0.001	THBS2	9.35 (4.39 to 14.31)	<0.001	0.01	TNFRSF11A	1.09 (0.31 to 1.88)	0.006	0.046
BNP	4.12 (2.36 to 5.88)	<0.001	<0.001	AGRP	5.22 (2.7 to 7.74)	<0.001	<0.001	TIE2	2.17 (0.61 to 3.72)	0.006	0.046
NTproBNP	4.77 (2.78 to 6.76)	<0.001	<0.001	CD4	5.01 (1.90 to 8.13)	0.002	0.02	SPON2	2.65 (0.84 to 4.47)	0.004	0.045
				BNP	3.79 (2.90 to 4.68)	<0.001	<0.001	THBS2	2.42 (0.69 to 4.14)	0.006	0.046
				NTproBNP	2.90 (1.84 to 3.96)	<0.001	<0.001	PRELP	2.78 (0.90 to 4.66)	0.004	0.045
								PTX3	1.14 (0.45 to 1.84)	0.001	0.02
								CTSL1	1.19 (0.35 to 2.03)	0.006	0.046
								CD4	1.76 (0.68 to 2.83)	0.001	0.02
								Troponin	0.95 (0.32 to 1.58)	0.003	0.04
								BNP	0.86 (0.53 to 1.18)	<0.001	<0.001
								NTproBNP	0.80 (0.43 to 1.16)	<0.001	<0.001

*Multivariable model was adjusted for age, sex, body mass index, diabetes, systolic blood pressure, estimated glomerular filtration rate and rheumatic arthritis disease duration.*

*KIM1, Kidney injury molecule 1; BMP9, Bone morphogenetic protein 9; BNP, B-type natriuretic peptide; NTproBNP, N-terminal pro b-type natriuretic peptide; SLAMF7, SLAM family member 7; IL4RA, Interleukin-4 receptor subunit alpha; THBS2, Thrombospondin-2; AGRP, Agouti-related protein; CD4, T-cell surface glycoprotein CD4; ADM, Adrenomedullin; PGF, Placenta growth factor; TNFRSF11A, Tumor necrosis factor receptor superfamily member 11A; TIE2, Angiopoietin-1 receptor; SPON2, Spondin-2; PRELP, Prolargin; PTX3, Pentraxin-related protein; CTSL1, Cathepsin L1*.

In the multivariable logistic regression analysis, higher levels of BNP/NT-proBNP were associated with cardiac structural and functional abnormalities (i.e., LVH, LAVi > 34 ml/m^2^, E/e′ > 14) and higher PGF level was associated with E/e′ > 14 (Supplementary Table 7).

Similar associations were found in population of patients with HFpEF and asymptomatic individuals (Supplementary Table 8).

### Echocardiographic Parameters, Circulating Biomarkers, and Clinical Outcomes

**Figure 1:**
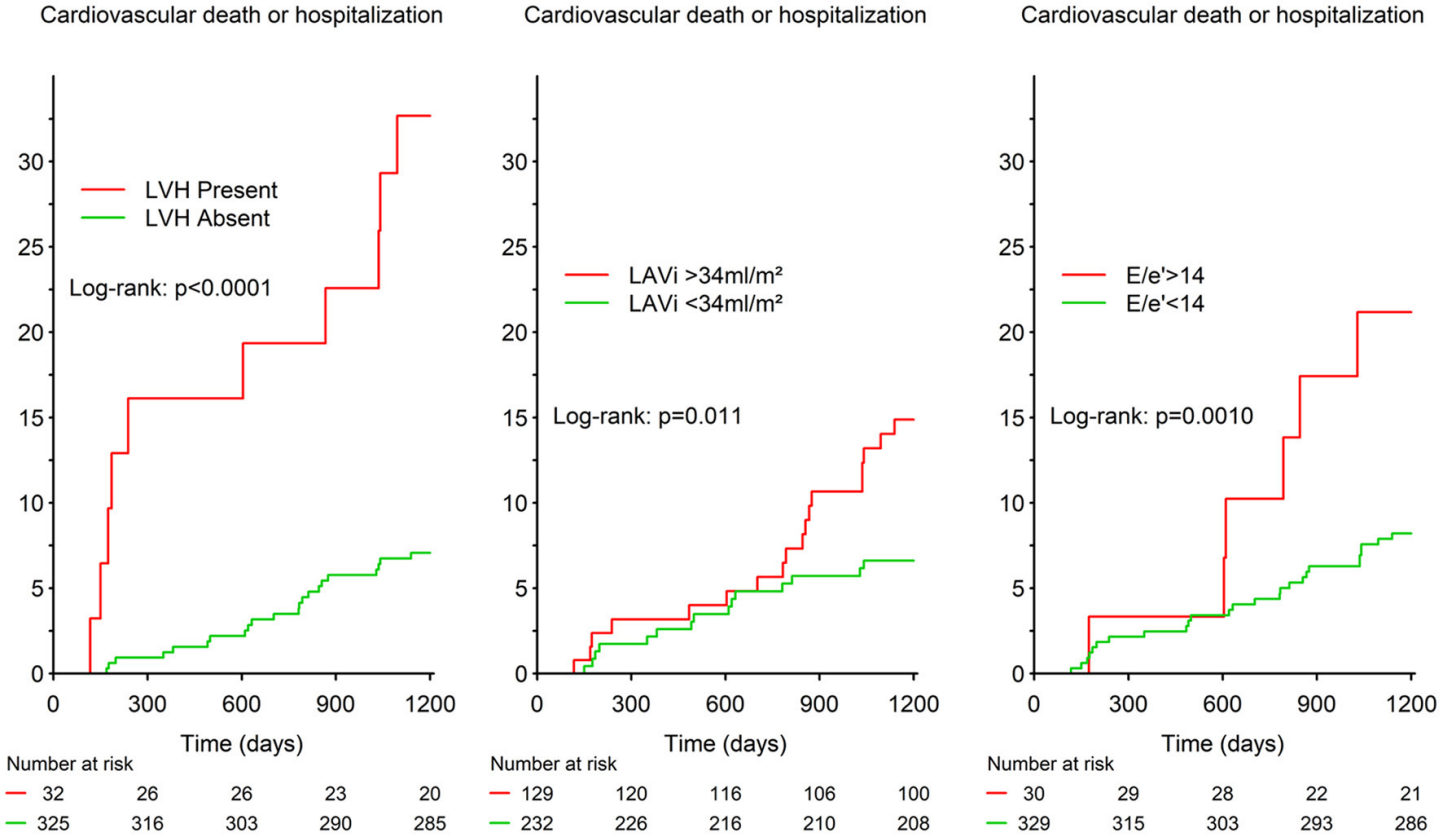
Survival curves for the primary outcome according to presence/absence of cardiac structure and function abnormalities in the RA Porto study. LVH, left ventricular hypertrophy; LAVi, left atrial volume index.

In patients with RA during a median follow-up of 48 months, the primary outcome (*n* = 37, 10.2%) occurred more frequently in patients with abnormalities of cardiac structure and function (Figure 1). In univariable model, greater LVMi [hazard ratio (HR) (95% CI) per 1 g/m^2^ = 1.03 (1.02–1.04), *p* < 0.001], greater LAVi [HR (95% CI) per 1 ml/m^2^ = 1.03 (1.01–1.06), *p* < 0.001], and higher E/e′ [HR (95% CI) per 1 = 1.08 (1.04–1.13), *p* < 0.001] were associated with higher rates of cardiovascular events. After adjustment for potential confounders, the poor prognosis associated with greater LVMi and higher E/e′ persisted (*p* < 0.05; Table 3).

**Table 3:** Survival analyses for the primary outcome in the RA Porto cohort.

	**Univariable model**		**Multivariable model**	
	**HR (95%CI)**	***p*-value**	**HR (95%CI)**	***p*-value**
LVMi (per 1 g/m^2^)	1.03 (1.02–1.04)	** <0.001**	1.03 (1.01–1.04)	** <0.001**
LVH	5.38 (2.65–10.94)	** <0.001**	4.55 (2.17–9.51)	** <0.001**
LAVi (per 1 ml/m^2^)	1.03 (1.01–1.06)	**0.004**	1.02 (0.99–1.04)	0.21
LAVi > 34 ml/m^2^	2.27 (1.19–4.33)	0.01	1.71 (0.88–3.32)	0.12
E/e′ (per 1)	1.08 (1.04–1.13)	** <0.001**	1.06 (1.002–1.13)	**0.042**
E/e′ mean >14	3.47 (1.58–7.61)	**0.002**	2.44 (1.03–5.77)	**0.042**

*Multivariable model included age, sex, body mass index, diabetes, systolic blood pressure, estimated glomerular filtration rate and rheumatoid arthritis duration.*

*LVMi, left ventricular mass indexed; LVH, left ventricular hypertrophy; LAVi, left atrial volume indexed*.

Furthermore, higher levels of TNF-related apoptosis-inducing ligand receptor 2 (TRAILR2) and CTSL1 were significantly associated with higher risk of the outcome after adjustment for echocardiographic parameters plus clinical confounders (Table 4).

**Table 4:** Associations of circulating biomarkers with the primary outcome in the RA Porto cohort.

	**LVMi model**			**LVH model**			**LAVi model**			**LAVi** **>** **34 model**			**E/e**^**′**^ **model**			**E/e**^**′**^**>** **14 model**		
	**HR**	***p*-value**	**FDR**	**HR**	***p*-value**	**FDR**	**HR**	***p*-value**	**FDR**	**HR**	***p*-value**	**FDR**	**HR**	***p*-value**	**FDR**	**HR**	***p*-value**	**FDR**
TRAILR2	1.59	0.004	0.21	1.65	<0.001	**0.033**	1.62	<0.001	**0.046**	1.65	<0.001	**0.025**	1.63	0.002	0.077	1.61	0.002	0.089
CTSL1	2.99	<0.001	**0.007**	3.52	<0.001	** <0.001**	3.40	<0.001	** <0.001**	3.73	<0.001	** <0.001**	3.20	<0.001	**0.002**	3.18	<0.001	**0.001**

*Multivariable model included age, sex, body mass index, diabetes, systolic blood pressure, estimated glomerular filtration rate and rheumatoid arthritis duration plus echocardiographic parameters.*

*LVMi, left ventricular mass index; LVH, left ventricular hypertrophy; LAVi, left atrial volume index; TRAILR2, TNF-related apoptosis-inducing ligand receptor 2; CTSL1, Cathepsin L1*.

## Discussion

This study shows that cardiac structure (i.e., LVMi and LAVi) and diastolic function (i.e., E/e′) of patients with RA were associated with circulating biomarkers reflecting inflammation (i.e., BMP9, PTX3, and TNFRSF11A), adverse extracellular matrix remodeling (i.e., PGF), congestion (i.e., NT-proBNP and ADM), and myocardial injury (i.e., hsTnT). Alterations of these echocardiographic parameters were associated with a worse prognosis. In addition, higher levels of inflammatory biomarkers (i.e., TRAILR2 and CTSL1) were associated with poorer prognosis in RA, independent of these cardiac alterations. These associations were also found in a population of patients with HFpEF and asymptomatic individuals. Together, these findings suggest that biomarker-guided screening may be relevant to detect cardiac structure and functional alterations in patients with RA, who are at a high risk of cardiovascular events.

The inflammatory biomarker associations described here make biologic and pathophysiological sense. We observed that a greater LVMi was associated with lower BMP9 levels and higher NT-proBNP and hsTnT levels. BMP9, a member of transforming growth factor-β1, as a potent anti-inflammatory cytokine, is expressed in the blood and heart tissues in HF. This protein inhibits cardiac fibrosis and reduces LV mass in an experimental model of HF (19). In addition, NT-proBNP and hsTnT are strongly associated with abnormalities of left ventricle and with cardiovascular morbidity and/or mortality even in pre-clinical settings (20, 21).

E/e′ reflects left ventricular filling pressures and carries relevant prognostic value (22, 23). Several biomarkers reflecting systemic inflammation (e.g., TNFRSF11A, PTX3, CTSL1, and CD4) were increased in patients with RA, which may reflect a link between RA-associated inflammation and diastolic dysfunction (7). Other biomarkers associated with higher E/e′ included BNP/NT-proBNP, hsTnT, ADM, PGF, SPON-2, and THBS2. As E/e′ reflects left ventricular filling pressures and diastolic stiffness, it is not surprising its association with these biomarkers. For example, ADM is a vasoactive peptide synthesized by endothelial and vascular smooth muscle cells, which is increased by endothelial dysfunction and volume overload (24). PGF, a member of the vascular endothelial growth factor family, is expressed in myocytes and is increased by pressure and volume overload (25). SPON-2 expression has been associated with inflammatory processes and cardiac fibrosis (26). THBS2 has been associated with the severity of congestion in HFpEF (27). Concordantly, LAVi, reflecting left ventricular filling pressure, was also associated with many inflammatory biomarkers (e.g., CD4 and IL4RA).

Interestingly, TRAILR2 and CTSL1 expressed inflammation and apoptosis and were related to inflammatory status in RA (28, 29). Therefore, our observations showing higher levels of these biomarkers were associated with a higher incident outcome that supported an adverse prognosis of enhanced levels of inflammatory biomarkers in patients with RA.

Several algorithms combining LVMi, LAVi, and E/e′ have been proposed for diagnosing or grading diastolic function (14, 30). Our findings suggest that inflammatory biomarkers and widely available biomarkers as BNP/NT-proBNP and hsTnT should be used for the screening of cardiac alterations in patients with RA and asymptomatic individuals. These biomarkers may be used for an early detection of asymptomatic cardiac dysfunction and prompt an adequate control of cardiovascular and RA-related risk factors (e.g., hypertension, diabetes, and RA activity).

## Limitations

This study had several limitations. First, all the three cohorts are observational cohort studies; thus, causality cannot be inferred. Second, some patients in the RA Porto cohort did not undergo echocardiogram and were excluded from the final analysis. Third, the RA Porto and the STANISLAS cohorts came from a single center and some of the findings may be influenced by local echocardiographers or practice patterns. Fourth, abnormalities of cardiac structure and function were reported to be independent of traditional cardiac risk factors and coronary artery disease in patients with RA (31, 32); however, cardiovascular comorbidities (i.e., coronary artery disease and HF) may influence the associations between echocardiographic parameters and biomarkers. We observed consistent associations between biomarkers and echocardiographic parameters across the different populations (i.e., patients with RA, those with HFpEF, and asymptomatic individuals) and biomarkers associated with study outcomes. However, external validation in other cohorts of patients with RA is needed.

## Conclusion

Circulating biomarkers reflecting inflammation, extracellular matrix inflammation, remodeling, congestion, and myocardial injury were associated with underlying alterations of cardiac structure and function. Biomarkers might be used for the screening of cardiac alterations in patients with RA.

## Data Availability Statement

The raw data supporting the conclusions of this article will be made available by the authors, without undue reservation.

## Ethics Statement

The studies involving human participants were reviewed and approved by Centro Hospitalar do Porto with the number 2016-023 (020-DEFI/020-CES). The patients/participants provided their written informed consent to participate in this study.

## Author Contributions

MK and MF: drafting. JF: supervision. JF, PRod, NG, PRos, and FZ: critical review of the manuscript. MF, RC, TF, JO, AM, and HC: data collection. MK and JF: analysis. All authors contributed to the article and approved the submitted version.

## Conflict of Interest

The authors declare that the research was conducted in the absence of any commercial or financial relationships that could be construed as a potential conflict of interest.

## Publisher's Note

All claims expressed in this article are solely those of the authors and do not necessarily represent those of their affiliated organizations, or those of the publisher, the editors and the reviewers. Any product that may be evaluated in this article, or claim that may be made by its manufacturer, is not guaranteed or endorsed by the publisher.

## References

[1] Gabriel SE Crowson CS Kremers HM Doran MF Turesson C O'Fallon WM. Survival in rheumatoid arthritis: a population-based analysis of trends over 40 years. Arthritis Rheum. (2003) 48:54–8. 10.1002/art.10705 12528103

[2] Mason JC Libby P Cardiovascular disease in patients with chronic inflammation: mechanisms underlying premature cardiovascular events in rheumatologic conditions. Eur Heart J. (2015) 36:482–9c. 10.1093/eurheartj/ehu403 25433021 PMC4340364

[3] Liang KP Myasoedova E Crowson CS Davis JM Roger VL Karon BL. Increased prevalence of diastolic dysfunction in rheumatoid arthritis. Ann Rheum Dis. (2010) 69:1665–70. 10.1136/ard.2009.124362 20498217 PMC2920362

[4] Packer M Lam CSP Lund LH Maurer MS Borlaug BA. Characterization of the inflammatory-metabolic phenotype of heart failure with a preserved ejection fraction: a hypothesis to explain influence of sex on the evolution and potential treatment of the disease. Eur J Heart Fail. (2020) 22:1551–67. 10.1002/ejhf.1902 32441863 PMC7687188

[5] Cesari M Penninx BW Newman AB Kritchevsky SB Nicklas BJ Sutton-Tyrrell K. Inflammatory markers and onset of cardiovascular events: results from the Health ABC study. Circulation. (2003) 108:2317–22. 10.1161/01.CIR.0000097109.90783.FC 14568895

[6] Zile MR Desantis SM Baicu CF Stroud RE Thompson SB McClure CD. Plasma biomarkers that reflect determinants of matrix composition identify the presence of left ventricular hypertrophy and diastolic heart failure. Circ Heart Fail. (2011) 4:246–56. 10.1161/CIRCHEARTFAILURE.110.958199 21350055 PMC4071931

[7] Paulus WJ Tschöpe C. A novel paradigm for heart failure with preserved ejection fraction: comorbidities drive myocardial dysfunction and remodeling through coronary microvascular endothelial inflammation. J Am Coll Cardiol. (2013) 62:263–71. 10.1016/j.jacc.2013.02.092 23684677

[8] Ferreira MB Fonseca T Costa R Marinhoc A Carvalho HC Oliveira JC. Prevalence, risk factors and proteomic bioprofiles associated with heart failure in rheumatoid arthritis: the RA-HF study. Eur J Intern Med. (2021) 85:41–9. 10.1016/j.ejim.2020.11.002 33162300

[9] Stienen S Ferreira JP Kobayashi M Preud'homme G Dobre D Machu JL. Enhanced clinical phenotyping by mechanistic bioprofiling in heart failure with preserved ejection fraction: insights from the MEDIA-DHF study (The Metabolic Road to Diastolic Heart Failure). Biomarkers. (2020) 25:201–11. 10.1080/1354750X.2020.1727015 32063068

[10] Ferreira JP Girerd N Bozec E Merckle L Pizard A Bouali S. Cohort profile: rationale and design of the fourth visit of the STANISLAS cohort: a familial longitudinal population-based cohort from the Nancy region of France. Int J Epidemiol. (2018) 47:395. 10.1093/ije/dyx240 29220499

[11] Villeneuve ENJ Emery P (2010). ACR-EULAR classification criteria for rheumatoid arthritis. Rev Bras Reumatol. (2010) 50:481–3. 10.1590/S0482-50042010000500001 21125184

[12] Lang RM BL Mor-Avi V Afilalo J Armstrong A Ernande L Flachskampf FA. Recommendations for cardiac chamber quantification by echocardiography in adults: an update from the American Society of Echocardiography and the European Association of Cardiovascular Imaging. Eur Heart J Cardiovasc Imaging. (2015) 16:233–70. 10.1093/ehjci/jev014 25712077

[13] Rudski LG Lai WW Afilalo J Hua L Handschumacher MD Chandrasekaran K. Guidelines for the echocardiographic assessment of the right heart in adults: a report from the American Society of Echocardiography endorsed by the European Association of Echocardiography, a registered branch of the European Society of Cardiology, and the Canadian Society of Echocardiography. J Am Soc Echocardiogr. (2010) 23:685–713; quiz 786–8. 10.1016/j.echo.2010.05.010 20620859

[14] Nagueh SF Smiseth OA Appleton CP Byrd BF III Dokainish H Edvardsen T. Recommendations for the evaluation of left ventricular diastolic function by echocardiography: an update from the American Society of Echocardiography and the European Association of Cardiovascular Imaging. J Am Soc Echocardiogr. (2016) 29:277–314. 10.1016/j.echo.2016.01.011 27037982

[15] Enroth S Maturi V Berggrund M Enroth SB Moustakas A Johansson A. Systemic and specific effects of antihypertensive and lipid-lowering medication on plasma protein biomarkers for cardiovascular diseases. Sci Rep. (2018) 8:5531. 10.1038/s41598-018-23860-y 29615742 PMC5882890

[16] Yavasoglu I Senturk T Onbasili A. Diastolic dysfunction in rheumatoid arthritis and duration of disease. Rheumatol Int. (2008) 29:113–4. 10.1007/s00296-008-0625-5 18496692

[17] Levey AS Stevens LA Schmid CH Zhang YL Castro AF III Feldman HI. A new equation to estimate glomerular filtration rate. Ann Intern Med. (2009) 150:604–12. 10.7326/0003-4819-150-9-200905050-00006 19414839 PMC2763564

[18] Boccard J Rutledge DN. A consensus orthogonal partial least squares discriminant analysis (OPLS-DA) strategy for multiblock Omics data fusion. Anal Chim Acta. (2013) 769:30–9. 10.1016/j.aca.2013.01.022 23498118

[19] Morine KJ Qiao X York S Natov PS Paruchuri V Zhang Y. Bone morphogenetic protein 9 reduces cardiac fibrosis and improves cardiac function in heart failure. Circulation. (2018) 138:513–26. 10.1161/CIRCULATIONAHA.117.031635 29487140 PMC6111008

[20] Lukowicz TV Fischer M Hense HW Döring A Stritzke J Riegger G. BNP as a marker of diastolic dysfunction in the general population: importance of left ventricular hypertrophy. Eur J Heart Fail. (2005) 7:525–31. 10.1016/j.ejheart.2004.12.010 15921790

[21] de Lemos JA Drazner MH Omland T Ayers CR Khera A Rohatgi A. Association of troponin T detected with a highly sensitive assay and cardiac structure and mortality risk in the general population. JAMA. (2010) 304:2503–12. 10.1001/jama.2010.1768 21139111 PMC5621378

[22] Shah AM Cikes M Prasad N Li G Getchevski S Claggett B. Echocardiographic features of patients with heart failure and preserved left ventricular ejection fraction. J Am Coll Cardiol. (2019) 74:2858–73. 10.1016/j.jacc.2019.09.063 31806129 PMC13537700

[23] Nagueh SF. Left ventricular diastolic function: understanding pathophysiology, diagnosis, and prognosis with echocardiography. JACC Cardiovasc Imaging. (2019) 13:228–44. 10.1016/j.jcmg.2018.10.038 30982669

[24] Voors AA Kremer D Geven C Ter Maaten JM Struck J Bergmann A. Adrenomedullin in heart failure: pathophysiology and therapeutic application. Eur J Heart Fail. (2019) 21:163–71. 10.1002/ejhf.1366 30592365 PMC6607488

[25] Ky B French B Ruparel K Sweitzer NK Fang JC Levy WC. The vascular marker soluble fms-like tyrosine kinase 1 is associated with disease severity and adverse outcomes in chronic heart failure. J Am Coll Cardiol. (2011) 58:386–94. 10.1016/j.jacc.2011.03.032 21757116 PMC3193932

[26] Dogan I Yetim M Dogan T Kayadibi H Yilmaz MB Eser B. Relation of serum spondin-2 levels with cardiac morphology and inflammatory parameters in hemodialysis patients. Int Urol Nephrol. (2018) 50:2091–7. 10.1007/s11255-018-1996-5 30276603

[27] Kimura Y Izumiya Y Hanatani S Yamamoto E Kusaka H Tokitsu T e al. High serum levels of thrombospondin-2 correlate with poor prognosis of patients with heart failure with preserved ejection fraction. Heart Vessels. (2016) 31:52–9. 10.1007/s00380-014-0571-y 25150586

[28] Cunnane G FitzGerald O Hummel KM Youssef PP Gay RE Gay S. Synovial tissue protease gene expression and joint erosions in early rheumatoid arthritis. Arth Rheum. (2001) 44:1744–53. 10.1002/1529-0131(200108)44:8<1744::AID-ART309>3.0.CO2-K 11508424

[29] Ichikawa K Liu W Fleck M Zhang H Zhao L Ohtsuka T. TRAIL-R2 (DR5) mediates apoptosis of synovial fibroblasts in rheumatoid arthritis. J Immunol. (2003) 171:1061–9. 10.4049/jimmunol.171.2.1061 12847280

[30] Paulus WJ Tschope C Sanderson JE Rusconi C Flachskampf FA Rademakers FE. How to diagnose diastolic heart failure: a consensus statement on the diagnosis of heart failure with normal left ventricular ejection fraction by the Heart Failure and Echocardiography Associations of the European Society of Cardiology. Eur Heart J. (2007) 28:2539–50. 10.1093/eurheartj/ehm037 17428822

[31] Davis JM III Lin G Oh JK Crowson CS Achenbach SJ Therneau TM. Five-year changes in cardiac structure and function in patients with rheumatoid arthritis compared with the general population. Int J Cardiol. (2017) 240:379–85. 10.1016/j.ijcard.2017.03.108 28427850 PMC5495559

[32] Mantel Ä Holmqvist M Andersson DC Lund LH Askling J. Association between rheumatoid arthritis and risk of ischemic and nonischemic heart failure. J Am Coll Cardiol. (2017) 69:1275–85. 10.1016/j.jacc.2016.12.033 28279294

