# Supplementary material for: Circulating Biomarkers and Cardiac Structure and Function in Rheumatoid Arthritis
Source: Front Cardiovasc Med. 2021 Nov 18;8:754784. doi: 10.3389/fcvm.2021.754784 (PMC8636810; doi:10.3389/fcvm.2021.754784)
Supplement: Supplementary file 1 [file Data_Sheet_1.docx]

**Supplemental Table 1. STROBE Statement—Checklist of items that should be included in reports of cohort studies**

|  | Item No | Recommendation |  |
| --- | --- | --- | --- |
| **Title and abstract** | 1 | (*a*) Indicate the study’s design with a commonly used term in the title or the abstract | Abstract |
|  |  | (*b*) Provide in the abstract an informative and balanced summary of what was done and what was found | Abstract |
| Introduction | | |  |
| Background/rationale | 2 | Explain the scientific background and rationale for the investigation being reported | Introduction paragraph 1&2 |
| Objectives | 3 | State specific objectives, including any prespecified hypotheses | Introduction paragraph 2 |
| Methods | | |  |
| Study design | 4 | Present key elements of study design early in the paper | Introduction paragraph 3 |
| Setting | 5 | Describe the setting, locations, and relevant dates, including periods of recruitment, exposure, follow-up, and data collection | Study population (Methods) paragraph 1 |
| Participants | 6 | (*a*) Give the eligibility criteria, and the sources and methods of selection of participants. Describe methods of follow-up | Study population (Methods) paragraph 1 |
|  |  | (*b*) For matched studies, give matching criteria and number of exposed and unexposed | Not applicable |
| Variables | 7 | Clearly define all outcomes, exposures, predictors, potential confounders, and effect modifiers. Give diagnostic criteria, if applicable | (Methods) Study population paragraph 1, Echocardiography, Circulating Biomarkers |
| Data sources/ measurement | 8* | For each variable of interest, give sources of data and details of methods of assessment (measurement). Describe comparability of assessment methods if there is more than one group | (Methods) Study population paragraph 1, Echocardiography, Circulating Biomarkers |
| Bias | 9 | Describe any efforts to address potential sources of bias | Not applicable |
| Study size | 10 | Explain how the study size was arrived at | Not applicable |
| Quantitative variables | 11 | Explain how quantitative variables were handled in the analyses. If applicable, describe which groupings were chosen and why | Statistical Analysis (Methods) |
| Statistical methods | 12 | (*a*) Describe all statistical methods, including those used to control for confounding | Statistical Analysis (Methods) |
|  |  | (*b*) Describe any methods used to examine subgroups and interactions | Not applicable |
|  |  | (*c*) Explain how missing data were addressed | Statistical Analysis (Methods) |
|  |  | (*d*) If applicable, explain how loss to follow-up was addressed | Not applicable |
|  |  | (*e*) Describe any sensitivity analyses | Not applicable |
| Results | | |  |
| Participants | 13* | (a) Report numbers of individuals at each stage of study—eg numbers potentially eligible, examined for eligibility, confirmed eligible, included in the study, completing follow-up, and analysed | Study population (Methods) |
|  |  | (b) Give reasons for non-participation at each stage | Study population (Methods) |
|  |  | (c) Consider use of a flow diagram | Not applicable |
| Descriptive data | 14* | (a) Give characteristics of study participants (eg demographic, clinical, social) and information on exposures and potential confounders | Baseline characteristics (Results) |
|  |  | (b) Indicate number of participants with missing data for each variable of interest | Not applicable |
|  |  | (c) summarize follow-up time (eg, average and total amount) | Study population (Methods) |
| Outcome data | 15* | Report numbers of outcome events or summary measures over time | Baseline Echocardiographic Parameters and Clinical Outcomes (Results) |
| Main results | 16 | (*a*) Give unadjusted estimates and, if applicable, confounder-adjusted estimates and their precision (eg, 95% confidence interval). Make clear which confounders were adjusted for and why they were included | Statistical Analysis (Methods) |
|  |  | (*b*) Report category boundaries when continuous variables were categorized | S Echocardiography (Methods) |
|  |  | (*c*) If relevant, consider translating estimates of relative risk into absolute risk for a meaningful time period | Not applicable |
| Other analyses | 17 | Report other analyses done—eg analyses of subgroups and interactions, and sensitivity analyses | Not applicable |
| Discussion | | |  |
| Key results | 18 | Summarize key results with reference to study objectives | Discussion paragraph 1 |
| Limitations | 19 | Discuss limitations of the study, taking into account sources of potential bias or imprecision. Discuss both direction and magnitude of any potential bias | Limitations (Discussion) |
| Interpretation | 20 | Give a cautious overall interpretation of results considering objectives, limitations, multiplicity of analyses, results from similar studies, and other relevant evidence | Discussion paragraph 2&3 |
| Generalizability | 21 | Discuss the generalizability (external validity) of the study results | Discussion paragraph 4 |
| Other information | | |  |
| Funding | 22 | Give the source of funding and the role of the funders for the present study and, if applicable, for the original study on which the present article is based | Funding |

**Supplemental table 2.** **Echocardiographic Parameters in the RA Porto cohort**

|  | **Intra-observer ICC** |
| --- | --- |
| **LV mass (g)** | 0.88 (0.69-0.94) |
| **LA volume (ml)** | 0.93 (0.85-0.98) |
| **E wave (m/s)** | 0.96 (0.87-0.98) |
| **Septal Ea Velocity (cm/s)** | 0.87 (0.74-0.93) |
| **Lateral Ea Velocity (cm/s)** | 0.95 (0.90-0.98) |
| **TR max velocity (m/sec)** | 0.83 (0.78-0.92) |

ICC, Intraclass Correlation Coefficients

**Supplemental table 3. Biomarker Names and Roles**

| **Biomarker full name** | **Entry name** | **Uniprot ID**** | **Angiogenesis** | **Blood vessel morphogenesis** | **Catabolic process** | **Cell adhesion** | **Coagulation** | **Heart development** | **Immune response** | **Inflammatory response** | **MAPK cascade** | **Platelet activation** | **Proteolysis** | **Regulation of blood pressure** | **Response to hypoxia** | **Response to peptide hormone** | **Wound healing** | **Others** |
| --- | --- | --- | --- | --- | --- | --- | --- | --- | --- | --- | --- | --- | --- | --- | --- | --- | --- | --- |
| Angiotensin-converting enzyme 2 | ACE2 | Q9BYF1 |  |  | 〇 |  |  |  |  | 〇 |  |  | 〇 | 〇 |  |  |  |  |
| Adisintegrin and metalloproteinase with thrombospondin motifs 13 | ADAMTS13 | Q76LX8 |  |  | 〇 | 〇 | 〇 |  | 〇 |  |  | 〇 | 〇 |  |  |  | 〇 |  |
| Adrenomedullin | ADM | P35318 | 〇 | 〇 |  |  |  | 〇 | 〇 |  |  |  |  |  | 〇 | 〇 |  |  |
| Agouti-related protein | AGRP | O00253 |  |  |  |  |  |  |  |  |  |  |  |  |  | 〇 |  |  |
| Protein AMBP | AMBP | P02760 |  |  | 〇 | 〇 |  |  | 〇 |  | 〇 |  |  |  |  |  |  |  |
| Angiopoietin-1 | ANG1 | Q15389 | 〇 | 〇 |  | 〇 |  |  | 〇 | 〇 | 〇 |  |  |  |  |  |  |  |
| Bone morphogenetic protein 6 | BMP6 | P22004 |  |  |  |  |  |  | 〇 | 〇 | 〇 |  |  |  |  |  |  |  |
| Natriuretic peptides B | BNP | P16860 | 〇 | 〇 |  |  |  |  |  |  |  |  |  | 〇 |  |  |  |  |
| Carbonic anhydrase 5A, mitochondrial | CA5A | P35218 |  |  |  |  |  |  |  |  |  |  |  |  |  |  |  | 〇 |
| C-C motif chemokine 17 | CCL17 | Q92583 |  |  |  |  |  |  | 〇 | 〇 | 〇 |  |  |  |  |  |  |  |
| C-C motif chemokine 3 | CCL3 | P10147 |  |  |  |  |  |  | 〇 | 〇 | 〇 |  |  |  |  |  |  |  |
| T-cell surface glycoprotein CD4 | CD4 | P01730 |  |  |  | 〇 |  |  | 〇 |  |  |  |  |  |  |  |  |  |
| CD40 ligand | CD40L | P29965 |  |  |  | 〇 | 〇 |  | 〇 | 〇 | 〇 | 〇 |  |  |  |  | 〇 |  |
| SLAM family member 5 | CD84 | Q9UIB8 |  |  |  | 〇 |  |  | 〇 |  |  |  |  |  |  |  |  |  |
| Carcinoembryonic antigenrelated cell adhesion molecule 8 | CEACAM8 | P31997 |  |  |  |  |  |  | 〇 |  |  |  |  |  |  |  |  |  |
| Chymotrypsin C | CTRC | Q99895 |  |  |  |  |  |  |  |  |  |  | 〇 |  |  |  |  |  |
| Cathepsin L1 | CTSL1 | P07711 |  |  | 〇 |  |  |  | 〇 |  |  |  | 〇 |  |  |  |  |  |
| C-X-C motif chemokine 1 (CVD2) | CXCL1 | P09341 |  |  |  |  |  |  | 〇 | 〇 |  |  |  |  |  |  |  |  |
| Decorin | DCN | P07585 | 〇 | 〇 | 〇 |  |  |  |  |  |  |  |  |  |  |  | 〇 |  |
| 2,4-dienoyl-CoA reductase, mitochondrial | DECR1 | Q16698 |  |  | 〇 |  |  |  |  |  |  |  |  |  |  |  |  |  |
| Dickkopf-related protein 1 | DKK1 | O94907 |  |  |  |  |  | 〇 |  |  |  |  |  |  |  |  |  |  |
| Fatty acid-binding protein, intestinal | FABP2 | P12104 |  |  | 〇 |  |  |  |  |  |  |  |  |  |  |  |  |  |
| Fibroblast growth factor 21 (CVD2) | FGF21 | Q9NSA1 |  |  |  |  |  |  |  |  | 〇 |  |  |  |  |  |  |  |
| Fibroblast growth factor 23 (CVD2) | FGF23 | Q9GZV9 |  |  | 〇 |  |  |  |  |  | 〇 |  |  |  |  |  |  |  |
| Follistatin | FS | P19883 |  |  |  |  |  |  |  |  |  |  |  |  |  |  |  | 〇 |
| Galectin-9 | GAL9 | O00182 |  |  |  | 〇 |  |  | 〇 | 〇 | 〇 |  | 〇 |  |  |  |  |  |
| Bone morphogenetic protein 9 (Growth/differentiation factor 2) | BMP9 or GDF2 | Q9UK05 | 〇 | 〇 |  |  |  |  |  |  | 〇 |  |  |  |  |  |  |  |
| Growth hormone | GH | P01241 |  |  |  |  |  |  |  |  | 〇 |  |  |  |  | 〇 |  |  |
| Gastric intrinsic factor | GIF | P27352 |  |  |  |  |  |  |  |  |  |  |  |  |  |  |  | 〇 |
| Lactoylglutathione lyase | GLO1 | Q04760 |  |  |  |  |  |  |  |  |  |  |  |  |  |  |  | 〇 |
| Gastrotropin | GT | P51161 |  |  | 〇 |  |  |  |  |  |  |  |  |  |  |  |  |  |
| Hydroxyacid oxidase 1 | HAOX1 | Q9UJM8 |  |  | 〇 |  |  |  |  |  |  |  |  |  |  |  |  |  |
| Proheparin-binding EGF-like growth factor | HBEGF | Q99075 |  |  |  |  |  |  |  |  | 〇 |  |  |  |  |  | 〇 |  |
| Heme oxygenase 1 | HO1 | P09601 | 〇 | 〇 | 〇 |  |  |  | 〇 | 〇 |  |  |  | 〇 | 〇 |  | 〇 |  |
| Osteoclast-associated immunoglobulin-like receptor | HOSCAR | Q8IYS5 |  |  |  |  |  |  | 〇 |  |  |  |  |  |  |  |  |  |
| Heat shock 27 kDa protein | HSP27 | P04792 | 〇 | 〇 | 〇 | 〇 | 〇 |  |  |  |  | 〇 |  |  |  |  | 〇 |  |
| Alpha-L-iduronidase | IDUA | P35475 |  |  | 〇 |  |  |  |  |  |  |  |  |  |  |  |  |  |
| Low affinity immunoglobulin gamma Fc region receptor II-b | IGGFCRECEPTORIIB | P31994 |  |  |  |  |  |  | 〇 |  |  |  |  |  |  |  |  |  |
| Pro-interleukin-16 | IL16 | Q14005 |  |  |  |  |  |  | 〇 |  | 〇 |  |  |  |  |  |  |  |
| Interleukin-17D | IL17D | Q8TAD2 |  |  |  |  |  |  |  | 〇 |  |  |  |  |  |  |  |  |
| Interleukin-18 (CVD2) | IL18 | Q14116 | 〇 | 〇 |  | 〇 |  |  | 〇 | 〇 | 〇 |  |  |  |  |  |  |  |
| Interleukin-1 receptor antagonist protein | IL1RA | P18510 |  |  |  | 〇 |  |  |  | 〇 |  |  |  |  |  |  |  |  |
| Interleukin-1 receptor-like 2 | IL1RL2 | Q9HB29 |  |  |  | 〇 |  |  | 〇 | 〇 |  |  |  |  |  |  |  |  |
| Interleukin-27 | IL27 | Q8NEV9 |  |  |  | 〇 |  |  | 〇 | 〇 |  |  |  |  |  |  |  |  |
| Interleukin-4 receptor subunit alpha | IL4RA | P24394 |  |  |  | 〇 |  |  | 〇 | 〇 |  |  |  |  |  |  |  |  |
| Interleukin-6 (CVD2) | IL6 | P05231 | 〇 | 〇 |  | 〇 | 〇 |  | 〇 | 〇 | 〇 | 〇 | 〇 |  |  | 〇 | 〇 |  |
| Melusin | ITGB1BP2 | Q9UKP3 |  |  |  |  |  |  |  |  |  |  |  |  |  |  |  | 〇 |
| Kidney injury molecule 1 | KIM1 | Q96D42 |  |  |  |  |  |  |  |  |  |  |  |  |  |  |  | 〇 |
| Leptin | LEP | P41159 | 〇 | 〇 | 〇 | 〇 |  |  | 〇 | 〇 | 〇 |  |  | 〇 | 〇 | 〇 |  |  |
| Lectin-like oxidized LDL receptor 1 | LOX1 | P78380 |  |  |  | 〇 |  |  |  | 〇 |  |  | 〇 |  |  |  |  |  |
| Lipoprotein lipase | LPL | P06858 |  |  | 〇 |  |  |  |  | 〇 |  |  |  |  |  |  |  |  |
| Macrophage receptor MARCO | MARCO | Q9UEW3 |  |  |  |  |  |  | 〇 |  |  |  |  |  |  |  |  |  |
| Tyrosine-protein kinase Mer | MERTK | Q12866 |  |  |  | 〇 | 〇 |  |  |  |  | 〇 |  |  |  |  | 〇 |  |
| Matrix metalloproteinase-12 | MMP12 | P39900 |  |  | 〇 |  |  |  |  |  |  |  | 〇 |  |  |  | 〇 |  |
| Matrix metalloproteinase-7 | MMP7 | P09237 |  |  | 〇 |  |  |  |  |  |  |  | 〇 |  |  |  |  |  |
| NF-kappa-B essential modulator | NEMO | Q9Y6K9 |  |  |  |  |  |  | 〇 | 〇 | 〇 |  |  |  |  |  |  |  |
| Pappalysin-1 | PAPPA | Q13219 |  |  |  |  |  |  |  |  |  |  |  |  |  |  |  | 〇 |
| Proteinase-activated receptor 1 | PAR1 | P25116 |  |  |  |  | 〇 |  |  | 〇 | 〇 | 〇 | 〇 | 〇 |  |  | 〇 |  |
| Poly [ADP-ribose] polymerase 1 | PARP1 | P09874 |  |  |  |  |  |  |  |  |  |  | 〇 |  |  | 〇 |  |  |
| Platelet-derived growth factor subunit B | PDGFSUBUNITB | P01127 |  |  |  |  | 〇 | 〇 |  |  | 〇 | 〇 |  | 〇 | 〇 | 〇 | 〇 |  |
| Programmed cell death 1 ligand 2 | PDL2 | Q9BQ51 |  |  |  | 〇 |  |  | 〇 |  |  |  |  |  |  |  |  |  |
| Polymeric immunoglobulin receptor | PIGR | P01833 |  |  |  |  |  |  | 〇 |  |  |  |  |  |  |  |  |  |
| Placenta growth factor | PLGF | P49763 | 〇 | 〇 |  |  |  |  |  |  |  |  |  |  | 〇 |  |  |  |
| Prolargin | PRELP | P51888 |  |  | 〇 |  |  |  |  |  |  |  |  |  |  |  |  |  |
| Brother of CDO | PROTEINBOC | Q9BWV1 |  |  | 〇 | 〇 |  |  |  |  |  |  |  |  |  |  |  |  |
| Serine protease 27 | PRSS27 | Q9BQR3 |  |  |  |  |  |  |  |  |  |  |  |  |  |  |  | 〇 |
| Prostasin | PRSS8 | Q16651 |  |  |  |  |  |  |  |  |  |  |  |  |  |  |  | 〇 |
| P-selectin glycoprotein ligand 1 | PSGL1 | Q14242 |  |  |  | 〇 |  |  |  |  |  |  |  |  |  |  |  |  |
| Pentraxin-related protein PTX3 | PTX3 | P26022 |  |  |  |  |  |  | 〇 | 〇 |  |  |  |  |  |  |  |  |
| Receptor for advanced glycosylation end products | RAGE | Q15109 |  |  | 〇 | 〇 |  |  | 〇 | 〇 |  |  |  |  |  |  |  |  |
| Renin | REN | P00797 |  |  |  |  |  |  |  |  | 〇 |  | 〇 | 〇 |  |  |  |  |
| Stem cell factor (CVD2) | SCF | P21583 |  |  |  | 〇 |  |  |  |  | 〇 |  |  |  |  |  |  |  |
| Serpin A12 | SERPINA12 | Q8IW75 |  |  |  |  |  |  |  |  |  |  |  |  |  | 〇 |  |  |
| SLAM family member 7 | SLAMF7 | Q9NQ25 |  |  |  | 〇 |  |  | 〇 |  |  |  |  |  |  |  |  |  |
| Superoxide dismutase [Mn], mitochondrial | SOD2 | P04179 |  |  |  |  |  |  |  |  |  |  |  | 〇 |  |  |  |  |
| Sortilin | SORT1 | Q99523 |  |  |  |  |  |  |  |  |  |  |  |  |  | 〇 |  |  |
| Spondin-2 | SPON2 | Q9BUD6 |  |  |  | 〇 |  |  | 〇 |  |  |  |  |  |  |  |  |  |
| Proto-oncogene tyrosine-protein kinase Src | SRC | P12931 |  |  |  | 〇 | 〇 |  | 〇 |  | 〇 | 〇 | 〇 |  | 〇 | 〇 | 〇 |  |
| Serine/threonine-protein kinase 4 | STK4 | Q13043 | 〇 | 〇 |  |  |  | 〇 |  |  |  |  |  |  |  |  |  |  |
| Tissue factor | TF | P13726 | 〇 | 〇 |  |  | 〇 |  |  | 〇 |  |  | 〇 |  |  |  | 〇 |  |
| Protein-glutamine gamma-glutamyltransferase 2 | TGM2 | P21980 |  |  |  | 〇 |  |  |  |  |  |  |  |  |  |  |  |  |
| Thrombospondin-2 | THBS2 | P35442 | 〇 | 〇 |  | 〇 |  |  |  |  |  |  |  |  |  |  |  |  |
| Thrombopoietin | THPO | P40225 |  |  |  |  |  |  |  |  | 〇 |  |  |  |  |  |  |  |
| Angiopoietin-1 receptor | TIE2 | Q02763 | 〇 | 〇 |  | 〇 |  | 〇 |  |  |  |  |  |  | 〇 | 〇 |  |  |
| Thrombomodulin | TM | P07204 |  |  |  |  | 〇 |  |  |  |  | 〇 |  |  |  |  | 〇 |  |
| Tumor necrosis factor receptor superfamily member 10A | TNFRSF10A | O00220 |  |  |  |  |  |  | 〇 | 〇 |  |  | 〇 |  |  |  |  |  |
| Tumor necrosis factor receptor superfamily member 11A | TNFRSF11A | Q9Y6Q6 |  |  |  |  |  |  | 〇 | 〇 | 〇 |  |  |  |  |  |  |  |
| Tumor necrosis factor receptor superfamily member 13B | TNFRSF13B | O14836 |  |  |  |  |  |  | 〇 |  |  |  |  |  |  |  |  |  |
| TNF-related apoptosis-inducing ligand receptor 2 | TRAILR2 | O14763 |  |  |  |  |  |  | 〇 | 〇 |  |  | 〇 |  |  |  |  |  |
| Vascular endothelial growth factor D | VEGFD | O43915 | 〇 | 〇 |  |  |  |  |  |  |  |  |  |  | 〇 |  |  |  |
| V-set and immunoglobulin domain-containing protein 2 | VSIG2 | Q96IQ7 |  |  |  |  |  |  |  |  |  |  |  |  |  |  |  | 〇 |
| Lymphotactin | XCL1 | P47992 |  |  |  | 〇 |  |  | 〇 | 〇 | 〇 |  |  |  |  |  |  |  |

*All biomarkers were sorted in alphabetical order.

Legend: *CVDII: cardiovascular II panel; **UniProt ID from UniProt Knowledgebase

**Supplemental table 4. Circulating Biomarkers Associated with Left Ventricular Mass Index**

| **biomarkers** | **beta (95%CI)** | **P** | **FDR** |  | **biomarkers** | **beta (95%CI)** | **P** | **FDR** |
| --- | --- | --- | --- | --- | --- | --- | --- | --- |
| **BMP6** | -2.01 (-6.52 to 2.51) | 0.38 | 0.75 |  | **TM** | -1.58 (-7.86 to 4.7) | 0.62 | 0.89 |
| **ANGPT1** | -0.37 (-2.77 to 2.03) | 0.76 | 0.91 |  | **VSIG2** | 0.52 (-2.63 to 3.67) | 0.75 | 0.91 |
| **ADM** | 5.14 (0.43 to 9.85) | **0.03** | 0.25 |  | **AMBP** | 9.06 (-1.21 to 19.32) | 0.08 | 0.39 |
| **CD40L** | -0.58 (-2.51 to 1.35) | 0.55 | 0.88 |  | **PRELP** | 2.10 (-8.17 to 12.37) | 0.69 | 0.91 |
| **SLAMF7** | 2.85 (-0.09 to 5.79) | 0.06 | 0.32 |  | **HO1** | 5.03 (0.41 to 9.66) | **0.03** | 0.25 |
| **PGF** | 6.21 (0.27 to 12.16) | **0.04** | 0.27 |  | **XCL1** | 1.19 (-1.76 to 4.13) | 0.43 | 0.81 |
| **ADAMTS13** | -2.4 (-17.15 to 12.35) | 0.75 | 0.91 |  | **IL16** | 1.37 (-2.74 to 5.48) | 0.51 | 0.88 |
| **BOC** | -5.43 (-12.65 to 1.78) | 0.14 | 0.48 |  | **SORT1** | 2.14 (-4.63 to 8.91) | 0.53 | 0.88 |
| **IL4RA** | 3.37 (-2.68 to 9.43) | 0.27 | 0.63 |  | **CEACAM8** | -0.30 (-3.58 to 2.98) | 0.86 | 0.93 |
| **SRC** | -2.09 (-7.30 to 3.11) | 0.43 | 0.81 |  | **PTX3** | 2.25 (-1.57 to 6.08) | 0.25 | 0.63 |
| **IL1ra** | 2.12 (-1.30 to 5.55) | 0.22 | 0.6 |  | **PSGL1** | -5.29 (-13.21 to 2.62) | 0.19 | 0.56 |
| **IL6** | 0.75 (-0.78 to 2.29) | 0.34 | 0.72 |  | **CCL17** | -0.74 (-2.82 to 1.34) | 0.48 | 0.87 |
| **TNFRSF10A** | 4.77 (-0.78 to 10.31) | 0.09 | 0.39 |  | **CCL3** | -0.02 (-2.76 to 2.72) | 0.99 | 0.99 |
| **STK4** | -2.4 (-5.41 to 0.61) | 0.12 | 0.43 |  | **MMP7** | 4.44 (0.82 to 8.06) | **0.02** | 0.22 |
| **IDUA** | -1.02 (-5.54 to 3.50) | 0.66 | 0.91 |  | **IgGFcreceptorIIb** | 0.25 (-1.83 to 2.33) | 0.81 | 0.91 |
| **TNFRSF11A** | 2.68 (-1.57 to 6.94) | 0.22 | 0.6 |  | **ITGB1BP2** | -0.30 (-2.03 to 1.44) | 0.74 | 0.91 |
| **PAR1** | -0.46 (-5.52 to 4.6) | 0.86 | 0.93 |  | **DCN** | -5.00 (-12.72 to 2.71) | 0.20 | 0.58 |
| **TRAILR2** | 4.20 (0.66 to 7.73) | **0.02** | 0.24 |  | **Dkk1** | 1.00 (-2.21 to 4.21) | 0.54 | 0.88 |
| **PRSS27** | 0.91 (-3.31 to 5.14) | 0.67 | 0.91 |  | **LPL** | -0.11 (-4.98 to 4.76) | 0.96 | 0.99 |
| **TIE2** | -6.10 (-14.56 to 2.37) | 0.16 | 0.49 |  | **PRSS8** | 6.13 (0.84 to 11.42) | **0.02** | 0.24 |
| **TF** | -0.16 (-6.42 to 6.10) | 0.96 | 0.99 |  | **AGRP** | 3.97 (-0.80 to 8.74) | 0.1 | 0.42 |
| **IL1RL2** | -1.28 (-5.6 to 3.05) | 0.56 | 0.88 |  | **HBEGF** | -0.99 (-4.12 to 2.15) | 0.54 | 0.88 |
| **PDGFsubunitB** | -0.67 (-3.26 to 1.92) | 0.61 | 0.89 |  | **BMP9** | -6.3 (-9.88 to -2.72) | **<0.001** | **0.01** |
| **IL27** | 4.92 (0.15 to 9.69) | **0.04** | 0.27 |  | **FABP2** | 1.00 (-1.24 to 3.25) | 0.38 | 0.75 |
| **IL17D** | -4.08 (-11.31 to 3.16) | 0.27 | 0.63 |  | **THPO** | 0.44 (-4.68 to 5.57) | 0.86 | 0.93 |
| **CXCL1** | -1.18 (-3.36 to 1.00) | 0.29 | 0.64 |  | **MARCO** | -0.63 (-10.29 to 9.03) | 0.90 | 0.95 |
| **LOX1** | -0.94 (-4.48 to 2.60) | 0.60 | 0.89 |  | **GT** | 2.98 (-0.21 to 6.18) | 0.07 | 0.33 |
| **Gal9** | 6.19 (-0.24 to 12.61) | 0.06 | 0.32 |  | **MMP12** | 0.52 (-2.04 to 3.08) | 0.69 | 0.91 |
| **GIF** | -0.01 (-1.58 to 1.56) | 0.99 | 0.99 |  | **ACE2** | 3.54 (0.38 to 6.70) | **0.03** | 0.25 |
| **SCF** | -3.94 (-8.82 to 0.94) | 0.11 | 0.43 |  | **PDL2** | -1.55 (-7.58 to 4.47) | 0.61 | 0.89 |
| **IL18** | 2.58 (-1.07 to 6.22) | 0.16 | 0.5 |  | **CTSL1** | 6.72 (2.18 to 11.27) | **<0.001** | 0.06 |
| **FGF21** | 1.1 (-0.05 to 2.25) | 0.06 | 0.32 |  | **hOSCAR** | -1.85 (-9.18 to 5.48) | 0.62 | 0.89 |
| **PIgR** | 6.77 (-8.02 to 21.57) | 0.37 | 0.75 |  | **TNFRSF13B** | 3.03 (-1.16 to 7.22) | 0.16 | 0.49 |
| **RAGE** | -5.20 (-10.00 to -0.40) | **0.03** | 0.25 |  | **TGM2** | -1.71 (-4.78 to 1.36) | 0.28 | 0.63 |
| **SOD2** | -4.33 (-23.29 to 14.64) | 0.65 | 0.91 |  | **LEP** | 0.36 (-2.41 to 3.13) | 0.8 | 0.91 |
| **CTRC** | -1.36 (-4.10 to 1.38) | 0.33 | 0.72 |  | **CA5A** | 1.09 (-1.32 to 3.50) | 0.37 | 0.75 |
| **FGF23** | 1.34 (-0.97 to 3.66) | 0.26 | 0.63 |  | **HSP27** | -2.73 (-11.51 to 6.04) | 0.54 | 0.88 |
| **SPON2** | 3.66 (-6.22 to 13.54) | 0.47 | 0.86 |  | **CD4** | 5.08 (-0.79 to 10.95) | 0.09 | 0.39 |
| **GH** | -0.09 (-1.16 to 0.98) | 0.87 | 0.93 |  | **NEMO** | -1.58 (-3.71 to 0.54) | 0.14 | 0.48 |
| **FS** | -0.43 (-3.80 to 2.95) | 0.80 | 0.91 |  | **VEGFD** | -1.08 (-7.20 to 5.04) | 0.73 | 0.91 |
| **GLO1** | -0.39 (-3.40 to 2.62) | 0.80 | 0.91 |  | **PARP1** | 2.21 (-1.55 to 5.96) | 0.25 | 0.63 |
| **CD84** | -1.56 (-6.45 to 3.32) | 0.53 | 0.88 |  | **HAOX1** | -0.23 (-1.58 to 1.12) | 0.74 | 0.91 |
| **PAPPA** | 0.52 (-2.90 to 3.94) | 0.76 | 0.91 |  | **Troponin** | 7.25 (3.87 to 10.62) | **<0.001** | **<0.001** |
| **SERPINA12** | 0.02 (-1.59 to 1.64) | 0.98 | 0.99 |  | **BNP** | 4.12 (2.36 to 5.88) | **<0.001** | **<0.001** |
| **REN** | -0.30 (-2.68 to 2.08) | 0.80 | 0.91 |  | **NTproBNP** | 4.77 (2.78 to 6.76) | **<0.001** | **<0.001** |
| **DECR1** | -1.43 (-3.18 to 0.31) | 0.11 | 0.42 |  |  |  |  |  |
| **MERTK** | -1.25 (-6.01 to 3.52) | 0.61 | 0.89 |  |  |  |  |  |
| **KIM1** | 4.41 (1.74 to 7.08) | **<0.001** | **0.02** |  |  |  |  |  |
| **THBS2** | -1.86 (-11.3 to 7.58) | 0.70 | 0.91 |  |  |  |  |  |

**Supplemental table 5. Circulating Biomarkers Associated with Left Atrial Volume Index**

| **biomarkers** | **beta (95%CI)** | **P** | **FDR** |  | **biomarkers** | **beta (95%CI)** | **P** | **FDR** |
| --- | --- | --- | --- | --- | --- | --- | --- | --- |
| **BMP6** | 0.47 (-1.94 to 2.88) | 0.70 | 0.81 |  | **TM** | -0.61 (-3.97 to 2.75) | 0.72 | 0.82 |
| **ANGPT1** | -0.90 (-2.18 to 0.37) | 0.16 | 0.38 |  | **VSIG2** | 0.51 (-1.18 to 2.21) | 0.55 | 0.74 |
| **ADM** | 3.36 (0.84 to 5.87) | **0.01** | 0.09 |  | **AMBP** | 5.69 (0.24 to 11.15) | **0.04** | 0.19 |
| **CD40L** | 0.13 (-0.89 to 1.16) | 0.8 | 0.87 |  | **PRELP** | 4.13 (-1.35 to 9.61) | 0.14 | 0.37 |
| **SLAMF7** | 2.59 (1.03 to 4.16) | **<0.001** | 0.02 |  | **HO1** | -1.76 (-4.24 to 0.72) | 0.16 | 0.38 |
| **PGF** | 2.62 (-0.58 to 5.82) | 0.11 | 0.37 |  | **XCL1** | 1.69 (0.12 to 3.26) | **0.04** | 0.18 |
| **ADAMTS13** | 8.61 (0.81 to 16.41) | **0.03** | 0.18 |  | **IL16** | 0.54 (-1.65 to 2.74) | 0.63 | 0.78 |
| **BOC** | -0.44 (-4.30 to 3.41) | 0.82 | 0.89 |  | **SORT1** | -0.91 (-4.54 to 2.71) | 0.62 | 0.78 |
| **IL4RA** | 5.71 (2.51 to 8.91) | **<0.001** | **0.01** |  | **CEACAM8** | -1.31 (-3.07 to 0.45) | 0.14 | 0.37 |
| **SRC** | 2.07 (-0.71 to 4.85) | 0.14 | 0.37 |  | **PTX3** | 1.90 (-0.14 to 3.94) | 0.07 | 0.28 |
| **IL1ra** | -2.01 (-3.84 to -0.17) | **0.03** | 0.18 |  | **PSGL1** | 1.39 (-2.85 to 5.64) | 0.52 | 0.74 |
| **IL6** | 0.07 (-0.76 to 0.89) | 0.88 | 0.93 |  | **CCL17** | -0.70 (-1.8 to 0.41) | 0.22 | 0.39 |
| **TNFRSF10A** | 3.44 (0.46 to 6.41) | **0.02** | 0.18 |  | **CCL3** | -0.60 (-2.06 to 0.87) | 0.42 | 0.66 |
| **STK4** | 0.41 (-1.22 to 2.03) | 0.62 | 0.78 |  | **MMP7** | 1.47 (-0.47 to 3.41) | 0.14 | 0.37 |
| **IDUA** | -1.84 (-4.25 to 0.57) | 0.13 | 0.37 |  | **IgGFcreceptorIIb** | 0.34 (-0.77 to 1.45) | 0.54 | 0.74 |
| **TNFRSF11A** | 1.99 (-0.29 to 4.28) | 0.09 | 0.33 |  | **ITGB1BP2** | 0.41 (-0.52 to 1.33) | 0.39 | 0.63 |
| **PAR1** | 3.01 (0.33 to 5.7) | **0.03** | 0.18 |  | **DCN** | 2.65 (-1.51 to 6.8) | 0.21 | 0.39 |
| **TRAILR2** | 0.66 (-1.25 to 2.58) | 0.5 | 0.73 |  | **Dkk1** | -0.38 (-2.09 to 1.33) | 0.66 | 0.78 |
| **PRSS27** | 2.19 (-0.04 to 4.42) | **0.05** | 0.24 |  | **LPL** | 0.42 (-2.16 to 3.00) | 0.75 | 0.84 |
| **TIE2** | 1.09 (-3.47 to 5.65) | 0.64 | 0.78 |  | **PRSS8** | -0.25 (-3.11 to 2.61) | 0.86 | 0.92 |
| **TF** | 2.54 (-0.81 to 5.89) | 0.14 | 0.37 |  | **AGRP** | 5.22 (2.70 to 7.74) | **<0.001** | **<0.001** |
| **IL1RL2** | -1.26 (-3.56 to 1.04) | 0.28 | 0.48 |  | **HBEGF** | -0.96 (-2.64 to 0.71) | 0.26 | 0.45 |
| **PDGFsubunitB** | -0.93 (-2.30 to 0.45) | 0.19 | 0.38 |  | **BMP9** | -0.62 (-2.57 to 1.33) | 0.53 | 0.74 |
| **IL27** | 1.65 (-0.92 to 4.22) | 0.21 | 0.39 |  | **FABP2** | 1.32 (0.13 to 2.52) | **0.03** | 0.18 |
| **IL17D** | 2.60 (-1.25 to 6.45) | 0.19 | 0.38 |  | **THPO** | 1.85 (-0.88 to 4.59) | 0.18 | 0.38 |
| **CXCL1** | 0.07 (-1.10 to 1.23) | 0.91 | 0.95 |  | **MARCO** | 3.99 (-1.16 to 9.14) | 0.13 | 0.37 |
| **LOX1** | -1.52 (-3.41 to 0.38) | 0.12 | 0.37 |  | **GT** | 1.09 (-0.63 to 2.81) | 0.21 | 0.39 |
| **Gal9** | 0.08 (-3.38 to 3.53) | 0.97 | 0.98 |  | **MMP12** | 0.99 (-0.38 to 2.36) | 0.15 | 0.38 |
| **GIF** | 0.19 (-0.65 to 1.03) | 0.66 | 0.78 |  | **ACE2** | 0.75 (-0.96 to 2.46) | 0.39 | 0.63 |
| **SCF** | -0.13 (-2.70 to 2.43) | 0.92 | 0.95 |  | **PDL2** | 1.86 (-1.37 to 5.08) | 0.26 | 0.45 |
| **IL18** | -1.41 (-3.36 to 0.54) | 0.16 | 0.38 |  | **CTSL1** | -0.32 (-2.78 to 2.15) | 0.80 | 0.87 |
| **FGF21** | 0.43 (-0.19 to 1.05) | 0.18 | 0.38 |  | **hOSCAR** | 1.53 (-2.41 to 5.46) | 0.45 | 0.69 |
| **PIgR** | 3.40 (-4.50 to 11.3) | 0.4 | 0.64 |  | **TNFRSF13B** | 3.05 (0.77 to 5.33) | 0.01 | 0.09 |
| **RAGE** | 2.04 (-0.54 to 4.62) | 0.12 | 0.37 |  | **TGM2** | -1.29 (-2.91 to 0.34) | 0.12 | 0.37 |
| **SOD2** | 6.62 (-3.53 to 16.77) | 0.2 | 0.39 |  | **LEP** | -1.29 (-2.77 to 0.19) | 0.09 | 0.33 |
| **CTRC** | -0.46 (-1.93 to 1.01) | 0.54 | 0.74 |  | **CA5A** | 0.93 (-0.36 to 2.22) | 0.16 | 0.38 |
| **FGF23** | 1.68 (0.44 to 2.91) | **0.01** | 0.09 |  | **HSP27** | 4.88 (0.20 to 9.57) | **0.04** | 0.19 |
| **SPON2** | 6.42 (1.15 to 11.7) | **0.02** | 0.15 |  | **CD4** | 5.01 (1.90 to 8.13) | **<0.001** | **0.02** |
| **GH** | 0.38 (-0.19 to 0.96) | 0.19 | 0.38 |  | **NEMO** | 0.37 (-0.77 to 1.51) | 0.52 | 0.74 |
| **FS** | 0.81 (-0.99 to 2.62) | 0.37 | 0.63 |  | **VEGFD** | 3.72 (0.48 to 6.96) | **0.03** | 0.18 |
| **GLO1** | -0.35 (-1.96 to 1.26) | 0.67 | 0.78 |  | **PARP1** | -0.61 (-2.62 to 1.41) | 0.56 | 0.74 |
| **CD84** | 0.75 (-1.86 to 3.35) | 0.57 | 0.75 |  | **HAOX1** | 0.16 (-0.57 to 0.88) | 0.67 | 0.78 |
| **PAPPA** | 0.02 (-1.8 to 1.85) | 0.98 | 0.98 |  | **Troponin** | 1.72 (-0.13 to 3.57) | 0.07 | 0.28 |
| **SERPINA12** | -0.56 (-1.41 to 0.3) | 0.20 | 0.39 |  | **BNP** | 3.79 (2.90 to 4.68) | **<0.001** | **<0.001** |
| **REN** | -0.27 (-1.55 to 1.00) | 0.67 | 0.78 |  | **NTproBNP** | 2.90 (1.84 to 3.96) | **<0.001** | **<0.001** |
| **DECR1** | 0.32 (-0.61 to 1.25) | 0.49 | 0.73 |  |  |  |  |  |
| **MERTK** | -0.07 (-2.63 to 2.49) | 0.96 | 0.98 |  |  |  |  |  |
| **KIM1** | 0.54 (-0.91 to 1.99) | 0.47 | 0.71 |  |  |  |  |  |
| **THBS2** | 9.35 (4.39 to 14.31) | **<0.001** | **0.01** |  |  |  |  |  |

**Supplemental table 6. Circulating Biomarkers Associated with E/e’**

| **biomarkers** | **beta (95%CI)** | **P** | **FDR** |  | **biomarkers** | **beta (95%CI)** | **P** | **FDR** |
| --- | --- | --- | --- | --- | --- | --- | --- | --- |
| **BMP6** | 1.11 (0.29 to 1.93) | **0.01** | 0.06 |  | **THBS2** | 2.42 (0.69 to 4.14) | **0.01** | 0.05 |
| **ANGPT1** | 0.12 (-0.32 to 0.56) | 0.6 | 0.71 |  | **TM** | 1.09 (-0.06 to 2.25) | 0.06 | 0.21 |
| **ADM** | 1.64 (0.77 to 2.51) | **<0.001** | **0.01** |  | **VSIG2** | 0.24 (-0.34 to 0.82) | 0.42 | 0.57 |
| **CD40L** | 0.18 (-0.17 to 0.54) | 0.31 | 0.48 |  | **AMBP** | 1.21 (-0.69 to 3.11) | 0.21 | 0.39 |
| **SLAMF7** | 0.45 (-0.1 to 0.99) | 0.11 | 0.3 |  | **PRELP** | 2.78 (0.9 to 4.66) | **<0.001** | **0.05** |
| **PGF** | 2.63 (1.56 to 3.70) | **<0.001** | **<0.001** |  | **HO1** | 0.82 (-0.04 to 1.68) | 0.06 | 0.21 |
| **ADAMTS13** | -1.95 (-4.65 to 0.76) | 0.16 | 0.33 |  | **XCL1** | 0.68 (0.14 to 1.23) | **0.01** | 0.09 |
| **BOC** | 0.96 (-0.37 to 2.28) | 0.16 | 0.33 |  | **IL16** | 0.42 (-0.34 to 1.18) | 0.28 | 0.45 |
| **IL4RA** | 0.58 (-0.53 to 1.7) | 0.31 | 0.48 |  | **SORT1** | 1.01 (-0.23 to 2.26) | 0.11 | 0.3 |
| **SRC** | 0.31 (-0.65 to 1.27) | 0.53 | 0.68 |  | **CEACAM8** | 0.34 (-0.27 to 0.95) | 0.28 | 0.45 |
| **IL1ra** | 0.44 (-0.2 to 1.08) | 0.18 | 0.36 |  | **PTX3** | 1.14 (0.45 to 1.84) | **<0.001** | **0.02** |
| **IL6** | 0.07 (-0.21 to 0.35) | 0.63 | 0.74 |  | **PSGL1** | 0.95 (-0.52 to 2.41) | 0.2 | 0.39 |
| **TNFRSF10A** | 1.02 (-0.01 to 2.05) | **0.05** | 0.19 |  | **CCL17** | 0.18 (-0.2 to 0.56) | 0.35 | 0.52 |
| **STK4** | 0.18 (-0.38 to 0.74) | 0.53 | 0.68 |  | **CCL3** | 0.17 (-0.33 to 0.68) | 0.5 | 0.66 |
| **IDUA** | -0.02 (-0.86 to 0.82) | 0.96 | 0.98 |  | **MMP7** | 0.01 (-0.66 to 0.68) | 0.98 | 0.98 |
| **TNFRSF11A** | 1.09 (0.31 to 1.88) | **0.01** | **0.05** |  | **IgGFcreceptorIIb** | -0.22 (-0.6 to 0.17) | 0.26 | 0.45 |
| **PAR1** | 0.69 (-0.24 to 1.62) | 0.15 | 0.33 |  | **ITGB1BP2** | 0.25 (-0.07 to 0.57) | 0.12 | 0.3 |
| **TRAILR2** | 0.52 (-0.13 to 1.18) | 0.12 | 0.3 |  | **DCN** | 1.11 (-0.31 to 2.54) | 0.13 | 0.3 |
| **PRSS27** | 0.61 (-0.16 to 1.38) | 0.12 | 0.3 |  | **Dkk1** | 0.35 (-0.24 to 0.94) | 0.24 | 0.43 |
| **TIE2** | 2.17 (0.61 to 3.72) | **0.01** | **0.05** |  | **LPL** | -0.82 (-1.71 to 0.07) | 0.07 | 0.22 |
| **TF** | 1.39 (0.24 to 2.53) | **0.02** | 0.11 |  | **PRSS8** | 0.43 (-0.55 to 1.42) | 0.39 | 0.55 |
| **IL1RL2** | 0.44 (-0.35 to 1.24) | 0.27 | 0.45 |  | **AGRP** | 0.51 (-0.38 to 1.39) | 0.26 | 0.45 |
| **PDGFsubunitB** | 0.15 (-0.33 to 0.62) | 0.54 | 0.69 |  | **HBEGF** | 0.09 (-0.49 to 0.67) | 0.76 | 0.86 |
| **IL27** | 0.88 (0 to 1.76) | **0.05** | 0.19 |  | **BMP9** | 0.2 (-0.48 to 0.87) | 0.57 | 0.7 |
| **IL17D** | 1.34 (0.01 to 2.67) | **0.05** | 0.19 |  | **FABP2** | 0.21 (-0.2 to 0.63) | 0.31 | 0.48 |
| **CXCL1** | -0.02 (-0.42 to 0.39) | 0.94 | 0.98 |  | **THPO** | 0.75 (-0.19 to 1.69) | 0.12 | 0.3 |
| **LOX1** | 0.04 (-0.61 to 0.7) | 0.9 | 0.97 |  | **MARCO** | 0.18 (-1.61 to 1.97) | 0.85 | 0.92 |
| **Gal9** | 1.37 (0.18 to 2.56) | **0.03** | 0.12 |  | **GT** | 0.18 (-0.42 to 0.77) | 0.55 | 0.69 |
| **GIF** | 0.01 (-0.28 to 0.3) | 0.92 | 0.98 |  | **MMP12** | 0.22 (-0.25 to 0.69) | 0.36 | 0.53 |
| **SCF** | 0.8 (-0.08 to 1.68) | 0.07 | 0.23 |  | **ACE2** | 0.27 (-0.32 to 0.85) | 0.37 | 0.54 |
| **IL18** | 0.72 (0.05 to 1.39) | 0.04 | 0.15 |  | **PDL2** | 0.44 (-0.68 to 1.55) | 0.44 | 0.59 |
| **FGF21** | -0.03 (-0.24 to 0.18) | 0.78 | 0.88 |  | **CTSL1** | 1.19 (0.35 to 2.03) | **0.01** | **0.05** |
| **PIgR** | -0.62 (-3.36 to 2.12) | 0.66 | 0.76 |  | **hOSCAR** | 1.05 (-0.32 to 2.41) | 0.13 | 0.31 |
| **RAGE** | -0.02 (-0.92 to 0.88) | 0.97 | 0.98 |  | **TNFRSF13B** | 0.46 (-0.32 to 1.24) | 0.25 | 0.44 |
| **SOD2** | -0.05 (-3.58 to 3.48) | 0.98 | 0.98 |  | **TGM2** | 0.36 (-0.21 to 0.92) | 0.21 | 0.39 |
| **CTRC** | 0.47 (-0.03 to 0.98) | 0.07 | 0.21 |  | **LEP** | 0.57 (0.06 to 1.07) | **0.03** | 0.14 |
| **FGF23** | 0.12 (-0.31 to 0.54) | 0.6 | 0.71 |  | **CA5A** | 0.31 (-0.13 to 0.76) | 0.17 | 0.34 |
| **SPON2** | 2.65 (0.84 to 4.47) | **<0.001** | **0.05** |  | **HSP27** | -0.09 (-1.71 to 1.54) | 0.92 | 0.98 |
| **GH** | 0.02 (-0.18 to 0.22) | 0.82 | 0.91 |  | **CD4** | 1.76 (0.68 to 2.83) | **<0.001** | **0.02** |
| **FS** | 0.17 (-0.45 to 0.79) | 0.59 | 0.71 |  | **NEMO** | 0.29 (-0.1 to 0.68) | 0.14 | 0.33 |
| **GLO1** | -0.23 (-0.78 to 0.33) | 0.42 | 0.57 |  | **VEGFD** | 1.31 (0.2 to 2.43) | 0.02 | 0.12 |
| **CD84** | 0.39 (-0.5 to 1.29) | 0.39 | 0.55 |  | **PARP1** | 0.76 (0.06 to 1.45) | 0.03 | 0.15 |
| **PAPPA** | 0.73 (0.1 to 1.35) | **0.02** | 0.12 |  | **HAOX1** | 0.05 (-0.2 to 0.3) | 0.69 | 0.79 |
| **SERPINA12** | 0.19 (-0.1 to 0.49) | 0.2 | 0.39 |  | **Troponin** | 0.95 (0.32 to 1.58) | **<0.001** | **0.04** |
| **REN** | 0.36 (-0.08 to 0.8) | 0.11 | 0.3 |  | **BNP** | 0.86 (0.53 to 1.18) | **<0.001** | **<0.001** |
| **DECR1** | 0.23 (-0.09 to 0.55) | 0.15 | 0.33 |  | **NTproBNP** | 0.8 (0.43 to 1.16) | **<0.001** | **<0.001** |
| **MERTK** | 0.4 (-0.48 to 1.29) | 0.37 | 0.54 |  |  |  |  |  |
| **KIM1** | 0.53 (0.01 to 1.04) | **0.05** | 0.18 |  |  |  |  |  |

**Supplemental table 7. Multivariable Selected Biomarkers for Each Echocardiographic Structural and Functional Abnormalities in the RA Porto cohort**

|  | **LVH** | | | **LAVi>34ml/m²** | | | **E/e'>14** | | |
| --- | --- | --- | --- | --- | --- | --- | --- | --- | --- |
|  | **OR** | **p-value** | **FDR** | **OR** | **p-value** | **FDR** | **OR** | **p-value** | **FDR** |
| **PGF** | 1.199 | 0.75 | 0.95 | 1.248 | 0.53 | 0.82 | 7.647 | <0.001 | **0.047** |
| **BNP** | 1.618 | <0.001 | 0.059 | 1.727 | <0.001 | **<0.001** | 1.586 | 0.003 | 0.068 |
| **NTproBNP** | 1.742 | 0.003 | 0.11 | 1.453 | 0.003 | 0.09 | 2.055 | 0.001 | **0.047** |

PGF, Placenta growth factor; BNP, B-type natriuretic peptide; NTproBNP, N-terminal pro b-type natriuretic peptide.

**Supplemental table 8. Multivariable Selected Biomarkers for Each Echocardiographic Parameter in the MEDIA-DHF and STANISLAS cohorts**

|  | MEDIA-DHF cohort | | | STANISLAS cohort | | |
| --- | --- | --- | --- | --- | --- | --- |
|  | **Biomarkers** | **beta (95%CI)** | **FDR** | **Biomarkers** | **beta (95%CI)** | **FDR** |
| LVMi (per 1g/m²) | **TNFRSF11A** | 11.61 (4.49 to 18.73) | 0.046 | **LEP** | -2.14 (-3.1 to -1.19) | <0.001 |
|  | **TM** | 22.92 (12.45 to 33.38) | 0.002 | **Troponin** | 1.57 (0.64 to 2.50) | 0.02 |
|  | **AMBP** | 32.71 (13.25 to 52.17) | 0.046 | **BNP** | 5.79 (3.98 to 7.60) | <0.001 |
|  |  |  |  | **NTproBNP** | 2.25 (1.31 to 3.19) | <0.001 |
| LAVi (per 1ml/m²) | **ADM** | 4.85 (1.78 to 7.92) | 0.02 | **ADM** | 1.57 (0.62 to 2.52) | 0.02 |
|  | **IL1RL2** | -5.88 (-9.73 to -2.03) | 0.03 | **FGF23** | 1.51 (0.65 to 2.37) | 0.01 |
|  | **RAGE** | 6.18 (2.54 to 9.81) | 0.01 | **PRELP** | 2.67 (0.87 to 4.47) | 0.04 |
|  | **FGF23** | 2.80 (1.36 to 4.23) | 0.003 | **XCL1** | 0.86 (0.27 to 1.46) | 0.045 |
|  | **THBS2** | 9.60 (3.28 to 15.93) | 0.03 | **DCN** | 2.55 (1.07 to 4.03) | 0.01 |
|  | **XCL1** | 3.71 (1.09 to 6.34) | 0.048 | **LPL** | 1.22 (0.43 to 2.00) | 0.03 |
|  | **MMP7** | 1.88 (0.75 to 3.01) | 0.02 | **Troponin** | 0.73 (0.33 to 1.13) | 0.01 |
|  | **LEP** | -4.92 (-7.11 to -2.72) | <0.001 | **BNP** | 4.32 (3.55 to 5.1) | <0.001 |
|  | **VEGFD** | 6.69 (2.86 to 10.53) | 0.01 | **NTproBNP** | 2.05 (1.65 to 2.46) | <0.001 |
|  | **BNP** | 3.48 (2.14 to 4.81) | <0.001 |  |  |  |
|  | **NTproBNP** | 5.19 (3.80 to 6.59) | <0.001 |  |  |  |
| E/e’ mean (per 1) | **ADM** | 1.47 (0.44 to 2.49) | 0.03 | **KIM1** | 0.21 (0.09 to 0.33) | 0.02 |
|  | **PGF** | 3.85 (2.37 to 5.34) | <0.001 | **LEP** | 0.15 (0.06 to 0.25) | 0.04 |
|  | **TNFRSF11A** | 2.25 (1.20 to 3.30) | 0.001 | **BNP** | 0.50 (0.32 to 0.68) | <0.001 |
|  | **TF** | 3.16 (1.47 to 4.84) | 0.003 | **NTproBNP** | 0.18 (0.09 to 0.27) | 0.01 |
|  | **IL27** | 2.65 (1.26 to 4.04) | 0.003 |  |  |  |
|  | **RAGE** | 1.73 (0.52 to 2.93) | 0.03 |  |  |  |
|  | **SPON2** | 4.08 (1.19 to 6.97) | 0.04 |  |  |  |
|  | **TM** | 2.50 (0.99 to 4.00) | 0.01 |  |  |  |
|  | **AGRP** | 2.01 (0.95 to 3.08) | 0.003 |  |  |  |
|  | **PDL2** | 2.29 (0.83 to 3.74) | 0.02 |  |  |  |
|  | **CTSL1** | 1.86 (0.74 to 2.98) | 0.01 |  |  |  |
|  | **TNFRSF13B** | 1.33 (0.42 to 2.23) | 0.03 |  |  |  |
|  | **CD4** | 2.27 (0.93 to 3.61) | 0.01 |  |  |  |
|  | **Troponin** | 0.68 (0.18 to 1.19) | 0.048 |  |  |  |
|  | **BNP** | 0.96 (0.50 to 1.41) | 0.001 |  |  |  |
|  | **NTproBNP** | 0.95 (0.46 to 1.44) | 0.003 |  |  |  |
